# Phase 1 study of the pan-HER inhibitor dacomitinib plus the MEK1/2 inhibitor PD-0325901 in patients with *KRAS-*mutation-positive colorectal, non-small-cell lung and pancreatic cancer

**DOI:** 10.1038/s41416-020-0776-z

**Published:** 2020-03-09

**Authors:** Robin M. J. M. van Geel, Emilie M. J. van Brummelen, Ferry A. L. M. Eskens, Sanne C. F. A. Huijberts, Filip Y. F. L. de Vos, Martijn P. J. K. Lolkema, Lot A. Devriese, Frans L. Opdam, Serena Marchetti, Neeltje Steeghs, Kim Monkhorst, Bas Thijssen, Hilde Rosing, Alwin D. R. Huitema, Jos H. Beijnen, René Bernards, Jan H. M. Schellens

**Affiliations:** 1 Department of Medical Oncology and Clinical Pharmacology, The Netherlands Cancer Institute, Amsterdam, Netherlands; 2 Department of Medical Oncology, Erasmus MC Cancer Institute, Rotterdam, Netherlands; 3 Department of Medical Oncology, UMC Utrecht Cancer Center, Utrecht, Netherlands; 4 Department of Pathology, The Netherlands Cancer Institute, Amsterdam, Netherlands; 5 Department of Pharmacy, The Netherlands Cancer Institute, Amsterdam, Netherlands; 6 Department of Clinical Pharmacy, University Medical Center Utrecht, Utrecht, Netherlands; 7 Utrecht Institute for Pharmaceutical Sciences, Utrecht University, Utrecht, Netherlands; 8 The Netherlands Cancer Institute, Division of Molecular Carcinogenesis & Oncode Institute, Amsterdam, Netherlands; 9 Faculty of science, Utrecht University, Utrecht, Netherlands; 10 Present Address: Maastricht University Medical Centre, Department of Clinical Pharmacy and Toxicology, Cardiovascular Research Institute Maastricht (CARIM), Maastricht, Netherlands; 11 Present Address: Centre for Human Drug Research, Leiden, Netherlands

**Keywords:** Colorectal cancer, Pancreatic cancer, Phase I trials, Non-small-cell lung cancer, Oncogenes

## Abstract

**Background:**

Mutations in *KRAS* result in a constitutively activated MAPK pathway. In *KRAS*-mutant tumours existing treatment options, e.g. MEK inhibition, have limited efficacy due to resistance through feedback activation of epidermal growth factor receptors (HER).

**Methods:**

In this Phase 1 study, the pan-HER inhibitor dacomitinib was combined with the MEK1/2 inhibitor PD-0325901 in patients with *KRAS*-mutant colorectal, pancreatic and non-small-cell lung cancer (NSCLC). Patients received escalating oral doses of once daily dacomitinib and twice daily PD-0325901 to determine the recommended Phase 2 dose (RP2D). (Clinicaltrials.gov: NCT02039336).

**Results:**

Eight out of 41 evaluable patients (27 colorectal cancer, 11 NSCLC and 3 pancreatic cancer) among 8 dose levels experienced dose-limiting toxicities. The RP2D with continuous dacomitinib dosing was 15 mg of dacomitinib plus 6 mg of PD-0325901 (21 days on/7 days off), but major toxicity, including rash (85%), diarrhoea (88%) and nausea (63%), precluded long-term treatment. Therefore, other intermittent schedules were explored, which only slightly improved toxicity. Tumour regression was seen in eight patients with the longest treatment duration (median 102 days) in NSCLC.

**Conclusions:**

Although preliminary signs of antitumour activity in NSCLC were seen, we do not recommend further exploration of this combination in *KRAS*-mutant patients due to its negative safety profile.

## Background

The RAS–RAF–MEK–ERK (MAPK) pathway plays a pivotal role in the regulation of cell proliferation, survival and differentiation. Persistent activation of this pathway is frequently observed in human cancers, and is associated with high rates of cancer cell proliferation. Commonly, pathway activation occurs as a consequence of oncogenic gain-of-function mutations in Kirsten rat sarcoma viral oncogene homolog (*KRAS*). The KRAS protein stimulates multiple downstream effector pathways, which are activated in a growth factor-independent way in cancer cells expressing oncogenic *KRAS.*^1–3^ The high frequency of *KRAS* mutations in human cancers (~20%) makes these proteins a potential target for antitumour therapy. The frequency of *KRAS* mutations is particularly high in pancreatic cancer (90%), colorectal cancer (CRC) (45%) and non-small-cell lung cancer (NSCLC) (35%).^1^

To date, with the exception of selective KRASG12C inhibitors such as AMG510,^4^ therapeutic approaches targeting and blocking KRAS directly have been unsuccessful. Small-molecule inhibitors against the downstream effectors of KRAS, such as MEK, demonstrated only limited antitumour activity in *KRAS*-mutated (*KRAS*m) cancers as well.^2,3,5,6^

Preclinical work from Sun and colleagues revealed that in *KRAS*m cancer cells, inhibition of MEK leads to feedback activation of upstream tyrosine kinase receptors, human epidermal growth factor receptor 2 (HER2) and 3 (HER3) in particular, causing intrinsic resistance through reactivation of the MAPK and phosphoinositide 3-kinase (PI3K) pathways.^7^ Concurrent treatment with a MEK inhibitor and an inhibitor of multiple HER receptor subtypes (pan-HER inhibitor) completely suppressed this feedback activation and resulted in synergistic antitumour activity in *KRAS*m cells in vitro and in xenograft models.^7^ As proof of concept was obtained in both *KRAS*m CRC and NSCLC models, we hypothesised that the antitumour activity of this approach would be independent of tumour histology. The unmet medical need for patients with *KRAS*m tumours and the high frequency of these mutations provided a rationale to investigate the combination of a MEK and pan-HER inhibitor in humans.

In this Phase 1 dose-finding study, we investigated the combination of dacomitinib, a potent irreversible ATP-competitive inhibitor of the HER kinase family (in vitro IC_50_ values of 6.0 nM, 45.7 nM and 74 nM against the human catalytic domains of HER1, HER2 and HER4), with PD-0325901, a highly specific non-ATP-competitive inhibitor of MEK1 and MEK2, in patients with *KRAS*m CRC, NSCLC or pancreatic cancer. The primary study objective was to determine the recommended phase 2 dose (RP2D) and schedule. The secondary objectives included characterising safety and tolerability, exploring antitumour activity and assessing the pharmacokinetic profiles of dacomitinib and PD-0325901 when given concomitantly.

## Methods

### Patient population

This investigator-initiated, multicentre, open-label, Phase 1 dose-escalation study enrolled patients at three sites in The Netherlands. Adult patients with histologically or cytologically confirmed advanced CRC, NSCLC or pancreatic cancer were enrolled on the basis of documented *KRAS* mutations in exons 2, 3 or 4, and *PIK3CA* wild-type status. Methods for analysing *KRAS* and *PIK3CA* status were analytically validated, and assessments were performed by a trained pathologist. *PIK3CA* wild type was required to avoid treatment resistance via activation of signalling proteins downstream of PIK3CA. Eligibility criteria included Eastern Cooperative Oncology Group (ECOG) performance status of <2, life expectancy of ≥3 months, measurable disease according to Response Evaluation Criteria in Solid Tumors (RECIST) version 1.1, adequate bone marrow (absolute neutrophil count ≥1.5 × 10^9^/L, platelets ≥100 × 10^9^/L and haemoglobin ≥6.0 mmol/L), hepatic (total bilirubin ≤1.5 × upper limit of normal [ULN], aspartate aminotransferase (AST) and alanine aminotransferase (ALT) ≤ 2.5 × ULN) and renal (serum creatinine ≤1.5 × ULN) functions. Radiotherapy, immunotherapy, chemotherapy or any treatment with investigational medication within 4 weeks prior to study treatment were not allowed, and patients with a history of other primary malignancies were excluded with the exception of patients who had been disease-free for ≥3 years, or with completely resected non-melanoma skin cancer. Additional exclusion criteria included symptomatic or untreated leptomeningeal disease, symptomatic brain metastasis, history of interstitial lung disease or pneumonitis, history of retinal vein occlusion and prior therapy containing targeted drug combinations known to interfere with EGFR, HER2, HER3, HER4 or MAPK- and PI3K-pathway components, including PI3K, AKT, mTOR, BRAF, MEK and ERK. The study was conducted in accordance with guidelines for Good Clinical Practice as defined by the International Conference on Harmonisation. Regulatory authorities and the institutional review boards approved the study protocol and all amendments. All patients gave written informed consent, per Declaration of Helsinki recommendations.

The study was registered at ClinicalTrials.gov (NCT02039336). Pfizer Inc. funded this study and provided the investigational drugs dacomitinib and PD-0325901.

### Study design and procedures

Patients were treated at varying dose levels of orally administered dacomitinib and PD-0325901 in cycles of 28 days. The starting doses were based on previous data from single-agent Phase 1 studies with both compounds, taking into account the potential for synergistic toxicity. Dose-level 1 consisted of 30 mg of dacomitinib once daily (QD) continuously, which is 67% of the maximum-tolerated dose and the recommended starting dose for EGFR-positive NSCLC as a single agent, and 2 mg of PD-0325901 twice daily (BID) administered on the first 21 days of each 28-day cycle, which is 25% of its single-agent-recommended dose. Subsequently, PD-0325901 was escalated according to a classical 3 + 3 design with fixed maximum escalation increments. Dose-escalation decisions were based on safety evaluation of all evaluable patients, performed after completion of the first treatment cycle. Patients were considered evaluable for the dose-determining part of this study if at least one cycle of study treatment was completed, with the minimum safety evaluation conducted, and at least one administration of both drugs received, or if dose-limiting toxicity (DLT) had occurred during the first cycle. If one out of three patients experienced a DLT, the number of patients treated at that dose level was expanded to a maximum of six. Dose escalation continued until a dose level was reached at which no more than one out of six patients experienced DLT during the first 28 days of treatment, provided that the single-agent-recommended doses of both compounds were not exceeded. Patients were continuing study treatment until disease progression, unacceptable toxicity or investigator/patient decision to discontinue.

Safety was monitored throughout the treatment by physical examination, laboratory assessments, electrocardiography, ophthalmic evaluation and collection of adverse events. Adverse events were recorded according to the Common Terminology Criteria for Adverse Events version 4.0. All adverse events that were possible, probable or definite related to study drug were considered as study/treatment related. DLT was defined as an adverse event or laboratory abnormality occurring within the first treatment cycle meeting at least one of the criteria described in supplementary table S1.

Radiologic tumour measurements were performed using computed tomography (CT) scans at baseline and every 6 weeks throughout the study. After a protocol amendment, the frequency was changed to every 8 weeks. Tumour response was evaluated according to RECIST 1.1.^8^ Patients were evaluable for antitumour activity if at least one follow-up radiologic evaluation was performed after the start of study treatment.

### Pharmacokinetic and pharmacodynamic analyses

For pharmacokinetic analyses, serial blood samples were obtained from all patients prior to treatment administration on day 1, and 1, 2, 3, 4, 6, 8, 12, 24, 72 and 144 h after the first dose. On day 1 of cycle 2, blood samples were drawn before and 1, 2, 3, 4, 6, 8, 12 and 24 h after administration. Plasma samples were assayed using a validated high-performance liquid chromatography– tandem mass spectrometry method (HPLC–MS/MS). Briefly, dacomitinib and PD-0325901 were extracted from plasma by protein precipitation with a mixture of acetonitrile/methanol (1:1 v/v). Compounds were chromatographically separated using a Waters Xbridge BEH Phenyl column (50 ×2.1-mm ID, 5-μm particle size), and detection was performed using an API4000 tandem mass spectrometer equipped with a turbo ion spray interface, operating in the positive ion mode. Transitions from *m*/*z* 480 to 329 and *m*/*z* 489 to 255 were monitored for the detection of dacomitinib and PD-0325901, respectively. Stable isotope-labelled internal standards were used for the quantification. The lower and upper limits of quantification were, respectively, 0.5 and 50 ng/ml for dacomitinib, and 5 and 500 ng/ml for PD-0325901. Pharmacokinetic parameters were calculated in R using an in-house-developed validated script for non-compartmental pharmacokinetic analyses (version 3.6.0).^9^

During the study, the protocol was amended to allow incorporation of tumour biopsies for pharmacodynamic analyses. Biopsies were taken before treatment, in the second week of treatment and upon treatment discontinuation. Phosphorylated (p)ERK and ribosomal pS6 (pS6-r) levels were assessed by validated immunohistochemistry (IHC) staining methods, and semi-quantitative H scores (percentage of positive cells (0–100) multiplied by staining intensity (0–3)) were assessed by an independent pathologist who was blinded for sample identification. Tumour biopsy samples were fixed in formalin for 16–24 h and embedded in paraffin subsequently. Immunohistochemistry of formalin-fixed paraffin-embedded tumour samples was performed on a BenchMark Ultra autostainer (Ventana Medical Systems). Briefly, paraffin sections were cut at 3 μm, heated at 75 °C for 28 min and deparaffinised in the instrument with EZ prep solution (Ventana Medical Systems). Heat-induced antigen retrieval was carried out using Cell Conditioning 1 (CC1, Ventana Medical Systems) at 95 °C for 32 and 64 min, for pS6-r and pERK1/2, respectively. pS6-r was detected using clone D68F8 (1:1000 dilution, 32 min at room temperature, Cell Signalling) and phospho-p44/42 MAPK (pERK1/2) (Thr202/Tyr204) using clone D13.14.4E (1:400 dilution, 1 h at room temperature, Cell Signalling). pERK was detected using the UltraView Universal DAB Detection Kit (Ventana Medical Systems), while detection of pS6-r was performed using the OptiView DAB Detection Kit (Ventana Medical Systems). Slides were counterstained with haematoxylin.

### Statistical analysis

Pharmacokinetics, pharmacodynamics, safety and tumour response data were reported descriptively.

## Results

### Patient disposition and characteristics

**Fig. 1:**
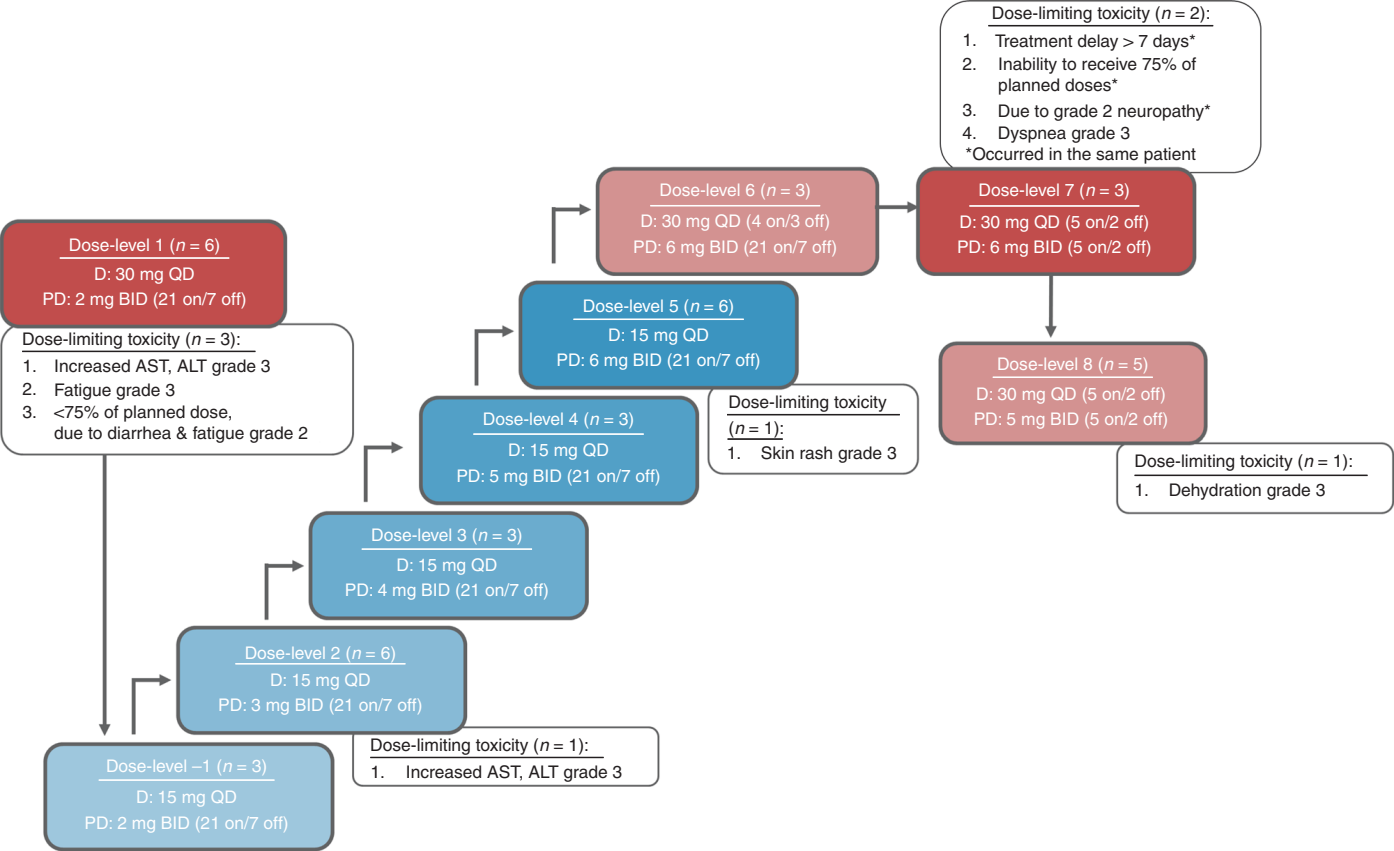
Dose-escalation cohorts and dose-limiting toxicities. D, dacomitinib; PD, PD-0325901; QD, once daily; BID, twice daily; AST, aspartate transaminase; ALT, alanine transaminase; n, number of patients; 21 on/7 off, 21 days on/7 days off; 4 on/3 off, 4 days on/3 days off; 5 on/2 off, 5 days on/2 days off.

Between April 2014 and April 2018, 41 patients (27 (66%) with CRC, 11 (27%) with NSCLC and 3 (7%) with pancreatic cancer) were enrolled into this study. The majority of patients had *KRAS* exon 2 mutations, and were pre-treated with at least two lines of antineoplastic therapy for advanced disease (Table 1). One patient with CRC did not wish to receive any antineoplastic therapy before enrolment, which was allowed per protocol. Thirty-eight patients were evaluable for dose determination (Fig. 1); three patients were considered not evaluable due to clinical deterioration, patient refusal and mistakenly administration of the wrong dose. At the end of the study, all (*n* = 41) patients had discontinued treatment due to progressive disease (*n* = 30), adverse events (*n* = 7), clinical deterioration/lack of benefit (*n* = 3) or patient refusal (*n* = 1).

**Table 1:** Patient and disease characteristics at baseline.

	Patients (*n* = 41)
Sex, *n* (%)	
Female	22 (54%)
Male	19 (46%)
Age, median (range), years	62 (43–81)
Tumour types, *n* (%)	
Colorectal	27 (66%)
Non-small-cell lung cancer	11 (27%)
Pancreatic	3 (7%)
ECOG PS, *n* (%)	
0	16 (39%)
1	25 (61%)
Number of prior lines of therapy, *n* (%)	
0	1 (2%)
1	7 (17%)
2	13 (32%)
≥3	20 (49%)
KRAS mutation, *n* (%)	
Exon 2	36 (88%)
Exon 3	3 (7%)
Exon 4	2 (5%)

*ECOG PS* Eastern Cooperative Oncology Group performance status, *KRAS* Kirsten rat sarcoma viral oncogene homologue.

### Dose finding

At the first dose level consisting of 30 mg of QD dacomitinib plus 2 mg of BID PD-0325901 (21 days on/7 days off), three out of six patients experienced DLTs, being grade 3-increased AST/ALT, grade 3 fatigue and inability to receive at least 75% of the planned dose due to grade 2 fatigue and diarrhoea (Fig. 1). Therefore, we decided to continue with a reduced dacomitinib dose of 15 mg in a continuous dosing schedule to allow for escalation of PD-0325901. In the subsequent dose levels with continuous administration of dacomitinib, DLTs were reported in two out of 21 patients: grade 3 AST/ALT increase (dose-level 2) and grade 3 skin rash (dose-level 5), respectively (Fig. 1). Although the formal RP2D was not reached, the escalation of PD-0325901 was halted in view of the increasing number of multiple grade 2 adverse events (e.g. diarrhoea, nausea and fatigue) beyond the DLT window of 28 days, together with the emergence of ocular toxicity at dose-level 5 (including retinopathy grade 1, retinal detachment grades 1 and 2 and dry eyes grade 1). The latter is known with the potential of more severe ocular toxicity at higher PD-0325901 doses.^10–12^ Consequently, the established maximum dose level with continuous dacomitinib dosing consisted of 15 mg of dacomitinib QD plus 6 mg of PD-0325901 BID. Subsequently, other intermittent regimens were initiated with the aim of optimising drug exposure and tolerability. A slight increase in exposure to dacomitinib was intended with dose-level 6 consisting of 30 mg of dacomitinib QD 4 days on/3 days off for 28 days, and PD-0325901 6 mg of BID for 21 days. No DLTs were observed at this dose level, which allowed further escalation of dacomitinib to 30 mg of QD 5 days on/2 days off. In view of patient convenience, it was decided to use a 5 day on/2 day off regimen for both agents, which should increase the exposure of dacomitinib with the same dose. Out of three patients, two experienced DLTs consisting of grade 2 neuropathy, leading to treatment delay of >7 days, and inability to receive 75% of the planned doses in one patient, and dyspnoea grade 3 in the other patient. This warranted dose de-escalation. Because a 5 day on/2 day off regimen was considered preferential, this regimen was maintained, and PD-0325901 was de-escalated to 5 mg of PD-0325901 BID combined with 30 mg of dacomitinib QD in dose-level 8. One DLT (dehydration grade 3) was observed, and apart from this event, the dose level was otherwise also not tolerable due to several grade 1 and 2 toxicities, most likely related to MEK inhibition with PD-0325901. The combination of dacomitinib and PD-0325901 was considered as too toxic and therefore not feasible in this relatively frail patient population.

### Safety

Study treatment-related adverse events were reported in all patients, with the most common being maculopapular and papulopustular rash (85%), diarrhoea (88%), nausea (63%), vomiting (41%) and fatigue (34%) (Table 2). Supportive care, including minocycline and cetomacrogol cream, or class I corticosteroid cream, was sufficient to manage skin rash, with the exception of one patient in dose-level 5 who had to discontinue treatment due to dose-limiting skin rash. The most frequent grade 3 events were diarrhoea (20%), nausea (12%) and fatigue (10%). Treatment interruption was caused by diarrhoea in five patients, by nausea in three patients, by rash in three patients and by fatigue in two patients. In all other cases, supportive care was sufficient to decrease the severity to grade 1 or less.

**Table 2:** Treatment-related adverse events, occurring in >10% of patients.

	Dose-level −1 (*n* = 4)		Dose-level 1 (*n* = 6)		Dose-level 2 (*n* = 6)		Dose-level 3 (*n* = 3)		Dose-level 4 (*n* = 3)		Dose-level 5 (*n* = 8)		Dose-level 6 (*n* = 3)		Dose-level 7 (*n* = 3)		Dose-level 8 (*n* = 5)		Total (*n* = 41)
Dacomitinib QD PD-0325901 BID	15 mg 2 mg		30 mg 2 mg		15 mg 3 mg		15 mg 4 mg		15 mg 5 mg		15 mg 6 mg		30 mg (4/3) 6 mg		30 mg (5/2) 6 mg (5/2)		30 mg (5/2) 5 mg (5/2)		All dose levels
Adverse event, *n* (%)	Gr 1/2	Gr 3	Gr 1/2	Gr 3	Gr 1/2	Gr 3	Gr 1/2	Gr 3	Gr 1/2	Gr 3	Gr 1/2	Gr 3	Gr 1/2	Gr 3	Gr 1/2	Gr 3	Gr 1/2	Gr 3	
Any skin toxicity^a^	3 (7%)		5 (12%)	1 (2%)	6 (15%)		3 (7%)		3 (7%)		5 (12%)	1 (2%)	3 (7%)		3 (7%)		4 (10%)		37 (90%)
Rash	3 (7%)		5 (12%)	1 (2%)	6 (15%)		3 (7%)		3 (7%)		4 (10%)	1 (2%)	3 (7%)		2 ((5%)		4 (10%)		35 (85%)
Dry skin			3 (7%)		1 (2%)						2 (5%)		1 (2%)		1 (2%)		1 (2%)		9 (22%)
Skin fissures			2 (5%)				2 (5%)												4 (10%)
PPE	1 (2%)				1 (2%)		1 (2%)		1 (2%)		2 (5%)								6 (15%)
Diarrhoea	4 (10%)		6 (15%)	2 (5%)	4 (10%)		1 (2%)	1 (2%)	2 (5%)		4 (10%)	3 (7%)	1 (2%)	1 (2%)	3 (7%)		3 (7%)	1 (2%)	36 (88%)
Nausea	4 (10%)		2 (5%)	1 (2%)	1 (2%)	1 (2%)	2 (5%)		2 (5%)	1 (2%)	5 (12%)	1 (2%)	2 (5%)				3 (7%)	1 (2%)	26 (63%)
Vomiting	3 (7%)		2 (5%)		1 (2%)	1 (2%)	2 (5%)		1 (2%)		5 (12%)	1 (2%)					1 (2%)		17 (41%)
Fatigue	1 (2%)		3 (7%)	1 (2%)	1 (2%)	1 (2%)	1 (2%)				3 (7%)			1 (2%)	1 (2%)			1 (2%)	14 (34%)
Anorexia	1 (2%)		3 (7%)		1 (2%)		1 (2%)		1 (2%)		3 (7%)						2 (5%)		12 (29%)
CPK increased			1 (2%)	1 (2%)	2 (5%)		1 (2%)		1 (2%)		2 (5%)		2 (5%)						10 (24%)
ALT/AST increased			3 (7%)	1 (2%)	1 (2%)	1 (2%)			1 (2%)		1 (2%)		1 (2%)						9 (22%)
Mucositis	2 (5%)		1 (2%)		1 (2%)		1 (2%)				2 (5%)		2 (5%)				2 (5%)		11 (27%)
Eye toxicity^b^			3 (7%)						1 (2%)		2 (5%)		2 (5%)						8 (20%)
Alopecia			3 (7%)		1 (2%)		1 (2%)				1 (2%)								5 (12%)
Dry mouth			2 (5%)		1 (2%)				1 (2%)		1 (2%)				1 (2%)				5 (12%)

All adverse events that are possible, probable or definite related to study drug were considered as study treatment related.

*QD* once daily, *BID* twice daily, *ALT/AST* alanine/aspartate transaminase, *4/3* 4 days on/3 days off, *5/2* 5 days on/2 days off, *CPK* creatine phosphokinase, *PPE* palmar plantar dysthesia syndrome.

^a^Some patients experienced one or more skin toxicities; only one was counted for the combined group of any toxicity.

^b^Includes neurosensory detachment, blurred vision, retinopathy, cataract and dry eyes.

Eye toxicities included grade 1 retinopathy, dry eyes grade 1, watering eyes grade 1, retinopathy grade 1 and retinal detachment grades 1 and 2 that occurred in four patients in dose levels 1, 5 and 6. All patients could continue study treatment without further progression of ocular toxicity. Cases of retinal vein occlusion were not observed in this study.

### Pharmacokinetic analysis

**Fig. 2:**
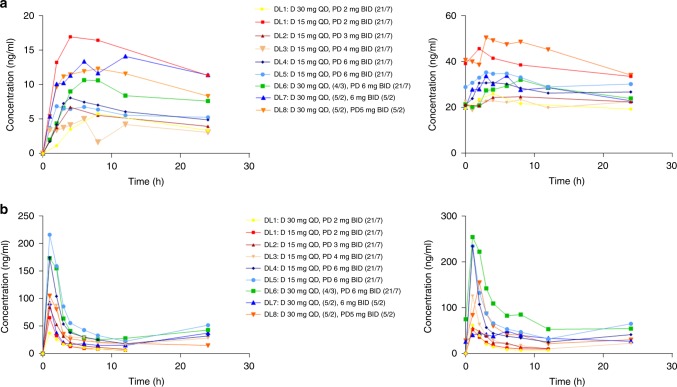
Pharmacokinetic profiles of dacomitinib and PD-0325901. All figures: plasma-concentration–time curves of mean plasma levels per dose level. DL, dose level; 21/7, 21 days on/7 days off; 4/3 4 days on/3 days off; 5/2 5 days on/2 days off. **a** Dacomitinib per dose level: cycle 1 day 1 (left) and cycle 2 day 1, or the last day of concomitant use of both drugs for intermittent dosing (right). **b** PD-0324901 per dose level: cycle 1 day 1 (left) and cycle 2 day 1, or the last day of concomitant use of both drugs for intermittent dosing (right).

Pharmacokinetic parameters after the first dose and at steady state are summarised in the supplementary data (Table S2). PD-0325901 and dacomitinib exposure increased approximately dose-proportionally with moderate and high inter-patient variability, respectively (Fig. 2). The half-life of dacomitinib could not be accurately calculated by non-compartmental analysis, due to its long terminal half-life. The long half-life and the high variability was known from previous studies, and was also reflected in our results.^12^ The mean dacomitinib peak plasma concentration (*C*_max_) and area under the plasma-concentration–time curve from time 0 to 24 h (AUC_0–24h_) increased approximately 3- to 5-fold after multiple dosing indicating extensive accumulation. A slight increase in AUC and *C*_max_ was also observed for PD-0325901 after multiple doses indicating minimal accumulation, which is in agreement with the relative short half-life (mean 7.7 h, range 5.0–9.9). Figure 2 shows the plasma-concentration–time curves per dose level.

### Antitumour activity

**Fig. 3:**
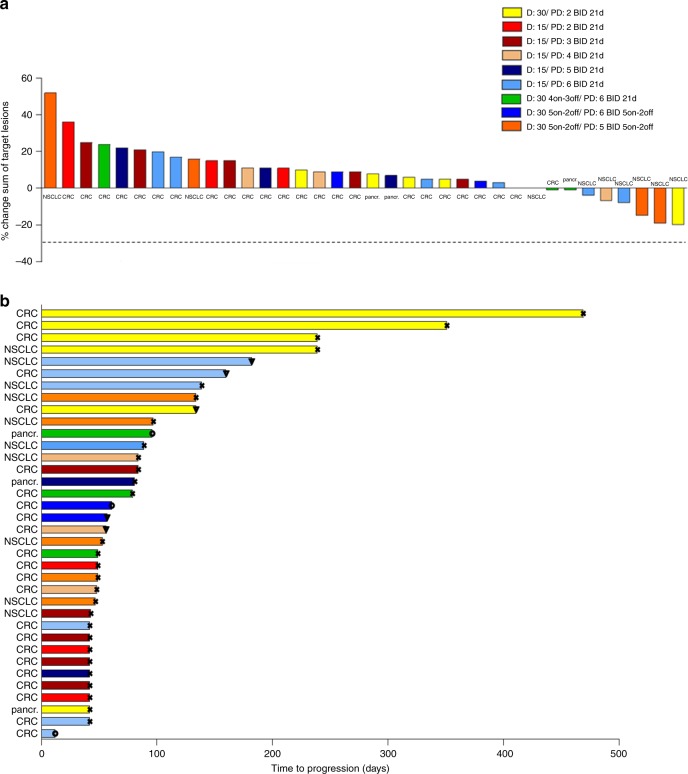
Antitumour activity of dacomitinib and PD-0325901 in *KRAS*m CRC, NSCLC and pancreatic cancer. **a** Maximum percentage change in the sum of target lesion size from baseline, including responses assessed by RECIST, by dose level. **b** Swimmer plot of treatment duration, by dose level. D, dacomitinib; PD, PD-0325901; BID, twice daily; CRC, colorectal cancer; NSCLC, non-small-cell lung cancer; pancr., pancreatic cancer; 21d, 21 days on/7 days off; 4on–3off, 4 days on/3 days off; 5on–2off, 5 days on/2 days off; PD, progressive disease. Symbols at the end of each bar represent the reason for the end of treatment for each individual patient.

Thirty-six patients were evaluable for antitumour activity (Fig. 3); five patients did not reach the first radiological evaluation due to clinical deterioration (*n* = 1), adverse events (*n* = 2), patient refusal (*n* = 1) or insufficient treatment (*n* = 1). Out of the evaluable patients, 20 achieved stable disease (including one patient with CRC and no prior treatment lines), and 16 had progressive disease on their first evaluation scan (*n* = 36). Tumour regression was seen in eight patients (18%) treated at various dose levels 1, 3, 5, 6 and 8 (Fig. 3a). Out of the eight evaluable patients with NSCLC, six achieved tumour regression within the limits of stable disease according to RECIST v1.1 criteria, and one had no change in target lesion volume as the best response. The overall median treatment duration was 90 days (range 3–469). Patients with NSCLC achieved the longest median treatment duration, 102 days (range 14–239), versus 87 days (range 3–469) for patients with CRC, and 73 days (42–96) for patients with pancreatic cancer. Median treatment duration was the longest in the dose levels that contained 30 mg of dacomitinib. In dose-level 1 with 30 mg of dacomitinib and 2 mg of PD-0325901, treatment duration was the longest (239 days, range 42–469), followed by dose-level 6 (dacomitinib 30 mg 4 days on/3 days off and PD-0325901 6 mg [79 days, range 49–96]) and 8 (dacomitinib 30 mg and PD-0325901 5 mg, both 5 days on/2 days off [77 days, range 43–134]) (Fig. 3b, *n* = 36).

### Pharmacodynamic analyses

Tumour biopsies were taken from seven patients at baseline and from four patients also on treatment (Fig. S1). In two patients from whom a paired biopsy was available, pERK was decreased, whereas from two other patients, pERK was increased during treatment. Reduction would be expected based on the mechanism of action of the drug combination. Only one of these two patients was evaluable for response and showed progressive disease. For one of the two patients with an increase in pERK, the biopsy was taken after 4 days of study treatment interruption, which may explain the lack of pERK modulation. In the other patient, the formalin fixation of the baseline biopsy was delayed, whereas direct fixation was desired. This delay might have caused degradation of phosphorylated proteins. These deviations will be discussed further in the next section.

Besides pERK staining, pS6 staining was also performed on tumour biopsies. However, pS6 staining results have to be interpreted with caution, because the quality of this staining could not be assured due to the lack of reliable controls. For this reason, pS6 staining results were not taken into account during the pharmacodynamic analyses of this clinical trial.

## Discussion

In this Phase 1 study, we investigated the combination of the MEK inhibitor PD-0325901 with the pan-HER inhibitor dacomitinib in patients with *KRAS*m NSCLC, CRC and pancreatic cancer. Based on preliminary efficacy results of the current trial and two comparable trials exploring the same treatment strategy, it was decided to limit recruitment to patients with *KRAS*m NSCLC in December 2016. At that moment, recruitment was ongoing in the dose-expansion cohort of dose-level 7.

Dose escalation was discontinued due to major toxicities in both continuous and intermittent dosing schedules. A rapid decline in performance status and poor overall tolerability played a major role in this. Furthermore, lack of efficacy was the second reason for the decision to discontinue enrolment.

Our study data showed that combining dacomitinib with PD-0325901 in a continuous or intermittent dosing schedule was not tolerated for the majority of patients. In a previous phase 1 dose-escalation study, PD-0325901 doses up to 20 mg of BID in a continuous dosing schedule, 30 mg of BID in a 21 day on/7 day off schedule and 10 mg in a 5 day on/2 day off schedule have been investigated. Although formal RP2Ds were established at 10 and 15 mg of BID in continuous and 5 day on/2 day off schedules, respectively, the occurrence of ocular toxicity, retinal vein occlusion in particular, decided us to reconsider the RP2D.^10^ As dacomitinib shows potential overlapping toxicity with PD-0325901, the starting doses for both agents were 25–70% of their monotherapy doses, being 2 mg of PD-0325901 BID in a 21 day on/7 day off schedule and 30 mg of dacomitinib QD. Although relatively low, these doses demonstrated target engagement and clinical activity in their respective single-agent studies.^10–12^ Nevertheless, the initial dose level was already not tolerated as indicated by DLTs in three out of six patients. Given the relatively low dose of PD-0325901 in relation to its single-agent maximum-tolerated dose, toxicity was likely to be associated with dacomitinib in particular. Therefore, the dacomitinib dose was reduced to enable dose escalation of PD-0325901, as we hypothesised that robust MEK inhibition was necessary to block the KRAS-activated MAPK pathway before tumour cells activate their escape mechanism through upstream tyrosine kinase receptors.^7^ Because ocular toxicity, i.e. asymptomatic central serous retinopathy, emerged at the 5 and 6mg dose levels, we halted dose escalation at 6 mg, and established the RP2D with continuous dacomitinib dosing at 15 mg of dacomitinib QD plus 6 mg of PD-0325901 BID 21 days on/7 days off. At doses of 5 and 6 mg, the plasma concentration of PD-0325901 exceeded the target level (16.5 ng/ml), consistent with target inhibition based on xenograft mouse models,^13^ during the entire dose interval (Fig. 2b). However, at 15 mg of dacomitinib doses, the plasma concentration did not exceed the preclinical target of 22 ng/ml, which is the IC_50_ for HER2/HER3 inhibition (unpublished data), for a substantial number of patients.

Therefore, after determination of the RP2D with continuous dacomitinib dosing, intermittent dosing schedules were initiated in an effort to optimise exposure and preserve tolerability. Dacomitinib, 30 mg of QD 4 days on/3 days off combined with PD-0325901 6 mg of BID 21 days on/7 days off, was better tolerated, but in view of therapy compliance, it was decided to further explore a 5 day on/2 day off regimen. Due to one DLT (dehydration grade 3) and multiple grade 1 and 2 toxicities in two additional patients, this dose level was considered intolerable. The combination of dacomitinib and PD-0325901 in a dose exceeding target levels was considered not manageable in these frail lung cancer patients.

Pharmacokinetic parameters of both agents were in line with previously reported single-agent data. Our data show no signs of pharmacokinetic interactions between the two agents.^10,12^

Unfortunately, pERK modulation in relation to tumour response data could only be assessed in one patient. Despite pERK reduction, this patient showed progressive disease. This could mean that target engagement was insufficient for antitumour response, in terms of duration or magnitude.

Patients with metastatic *KRAS*m tumours represent a population with a high unmet medical need. Multiple strategies to target KRAS have been explored, including farnesyltransferase inhibitors, small molecules interfering with the prenyl-binding protein PDEδ-KRAS interaction and small molecules targeting downstream effectors of KRAS, e.g. RAF, MEK or PI3K. However, none of these approaches have been successful.^2,14,15^ Since all these strategies rely on targeting a single protein or pathway, rapid onset of resistance due to tumour escape mechanisms exploiting alternative pathways is to be expected.^16^ Therefore, combination strategies may have a more sustained antitumour effect. Previously, van Geel et al. demonstrated clinical proof of concept for combining BRAF and EGFR inhibition in patients with *BRAF*-mutant CRC,^17^ based on a synthetic lethality drug screen.^18^ The BEACON CRC phase 3 trial investigating the combination of BRAF and EGFR inhibition with or without a MEK inhibitor also showed favourable results.^19^ Similarly, in *KRAS*m cells, inhibition of MEK was found to synergise with HER2 and HER3 inhibition in an identical screen to identify synthetic lethal interactions.^7^ However, in contrast to these preclinical observations, the preliminary clinical activity with dacomitinib plus PD-0325901 in *KRAS*m tumours was relatively disappointing. Toxicity restricted combining full single-agent doses, leading to a lower exposure, which potentially limits clinical antitumour activity.

Another explanation for the limited antitumour activity of this combination may lie in the extensive inter-pathway connections of the KRAS protein. Although we excluded patients with concurrent *KRAS* and *PIK3CA* mutations, activation of the PI3K pathway may as well be triggered by mutated *KRAS* directly, particularly in the presence of downstream MEK inhibition.^20^ In addition, reactivation of the MAPK pathway may occur as well, analogous to the observation with *BRAF* inhibition in *BRAF*-mutant CRC cells,^21^ especially when upstream receptors are not adequately inhibited. Indeed, although this concerns a small cohort, patients treated with doses of 30 mg of dacomitinib had disease stabilisation for a longer period of time compared with patients on dose levels containing 15 mg of dacomitinib (Fig. 3b).

Interestingly, six out of eight patients (75%) with NSCLC achieved tumour regression, compared with one out of 24 patients (4%) with CRC (Fig. 3a). In addition, the median treatment duration in patients with NSCLC (102 days) was longer than that of CRC patients (87 days), suggesting a difference in sensitivity to study treatment between these malignancies (Fig. 3b). This finding was also reflected in the results of two separate studies. Höchster et al. showed that adding a MEK inhibitor to second-line irinotecan therapy in patients with *KRAS*m CRC did not result in clinical benefit.^22^ However, patients with *KRAS*m NSCLC had an improved response rate by the addition of the MEK inhibitor selumetinib to second-line treatment with docetaxel as reported by Jänne et al., although no significant effect on progression-free and overall survival was observed.^5^

To explain the differences in sensitivity between tumour types, several biomarkers will be explored in a translational study on paired tumour biopsies from patients with *KRAS*m tumours treated with three different combinations of pan-HER and MEK inhibitors in this phase 1 trial and two other clinical trials. In the translational study, analyses will include at least the following biomarkers: HER3, heregulin, BCL-XL, *KRAS* mutant to *KRAS* wild-type allele frequency ratio, *KRAS* copy number, KRAS expression levels and the nature of the *KRAS* mutation.

In conclusion, dacomitinib could only be combined safely with PD-0325901 in a continuous or intermittent dosing schedule at doses much lower than the recommended single-agent doses. Toxicity prevented continuous dosing of dacomitinib and PD-0325901. Although preliminary signs of antitumour activity in NSCLC were seen, defining a dose and regimen with manageable (long-term) toxicity was not feasible. Therefore, it is not recommended to further explore the combination of dacomitinib and PD-0325901 in *KRAS*m NSCLC patients.

## Supplementary information

Table S1

Table S2

Figure S1

## Data Availability

The clinical datasets analysed during this clinical trial are available from the corresponding author on reasonable request, and only if anonymisation of patient data can be fully ensured.
