# Supplementary material for: Phase 1 study of the pan-HER inhibitor dacomitinib plus the MEK1/2 inhibitor PD-0325901 in patients with KRAS-mutation-positive colorectal, non-small-cell lung and pancreatic cancer
Source: Br J Cancer. 2020 Mar 9;122(8):1166–74. doi: 10.1038/s41416-020-0776-z (PMC7156736; doi:10.1038/s41416-020-0776-z)
Supplement: Supplementary file 1 — Table S1 [file 41416_2020_776_MOESM1_ESM.docx]

**Supplementary data
Table S1. Criteria for defining dose-limiting toxicities in 4 categories, including hematologic, non-hematologic, cardiac and other toxicities.***Abbreviations: AST, aspartate aminotransferase; AL,T alanine aminotransferase; ULN, upper limit of normal; LLN, lower limit of normal; DLT, dose limiting toxicity*

| **Toxicity** | **DLT definition** |
| --- | --- |
| Hematologic | - Grade 4 neutropenia for ≥ 5 days - Grade ≥ 3 febrile neutropenia - Grade 4 anemia - Grade 4 thrombocytopenia |
| Non-hematologic | - AST > 5X ULN OR, ALT > 3X ULN AND bilirubin > 2X ULN (after exclusion of disease progression and/or bile duct obstruction) - Grade ≥ 4 rash, hand-foot syndrome or photosensitivity - Grade 3 rash, hand-foot syndrome or photosensitivity for > 7 days despite adequate supportive treatment. - Grade ≥ 3 nausea, vomiting or diarrhea in the presence of maximal supportive care - Grade ≥ 2 peripheral sensory or motor neuropathy - Grade ≥ 3 clinically significant non-hematologic toxicity other than those listed above, with the following exceptions:   - Electrolyte disturbances that respond to correction within 24 hours   - Grade 3 hypertension that is adequately controlled by the addition of up to 2 additional antihypertensive medications   - Grade 3 pyrexia that does not result in study discontinuation |
| Cardiac | - Ejection fraction < lower limit of normal (LLN) with an absolute decrease of >10% from baseline with confirmation within 14 days |
| Other | - Inability to receive ≥75% of scheduled doses in treatment period due to toxicity related to study treatment - Treatment delay of > 7 days due to study treatment-related toxicity - Grade ≥2 toxicity that occurs beyond 28 days which in the judgment of the investigator is a DLT |
