# Supplementary material for: Phase 1 study of the pan-HER inhibitor dacomitinib plus the MEK1/2 inhibitor PD-0325901 in patients with KRAS-mutation-positive colorectal, non-small-cell lung and pancreatic cancer
Source: Br J Cancer. 2020 Mar 9;122(8):1166–74. doi: 10.1038/s41416-020-0776-z (PMC7156736; doi:10.1038/s41416-020-0776-z)
Supplement: Supplementary file 2 — Table S2 [file 41416_2020_776_MOESM2_ESM.docx]

**Table S2. Pharmacokinetic parameters of dacomitinib and PD-0325901, at baseline and steady-state, per dose-level***Abbreviations: C_max_, peak plasma concentration; t_max_, time of maximum plasma concentration observed; AUC_0-24h_, area under the plasma concentration-time curve from time zero to 24 hours; T_1/2_, elimination half-life; N/C, could not be calculated; 4/3, 4 days on / 3 days of; 5/2, 5 days on/2 days off*

| **Dose-level** | **1** | **-1** | **2** | **3** | **4** | **5** | **6** | **7** | **8** |  |  |  |  |  |  |  |
| --- | --- | --- | --- | --- | --- | --- | --- | --- | --- | --- | --- | --- | --- | --- | --- | --- |
| *Dacomitinib QD* | 30 mg | 15 mg | 15 mg | 15 mg | 15 mg | 15 mg | 30 mg (4/3) | 30 mg (5/2) | 30 mg (5/2) |  |  |  |  |  |  |  |
| *PD-0325901 BID* | 2 mg | 2 mg | 3 mg | 4 mg | 5 mg | 6 mg | 6 mg | 6 mg (5/2) | 5 mg (5/2) |  |  |  |  |  |  |  |
| **Dacomitinib** | *Cycle 1 Day 1* | | | | | |  |  |  | *All 15 mg*  *doses* | *All 30 mg*  *doses* |  |  |  |  |  |
| *Mean (CV%)* | n = 6 | n = 4 | n = 6 | n = 2 | n = 3 | n = 8 | n = 3 | n = 3 | n = 5 | n = 23 | n = 17 |  |  |  |  |  |
| C_max_ (ng/ml) | 19.2 (52) | 7.9 (43) | 6.7 (61) | 2.5 (48) | 8.5 (11) | 11.4 (74) | 11.1 (23) | 19.1 (38) | 14.2 (19) | 8.6 (69) | 16.0 (44) |  |  |  |  |  |
| t_max_ (h) | 7.5 | 5.6 | 8.7 | 12 | 4.7 | 7.9 | 7.3 | 13.3 | 6.6 | 7.6 | 8.3 |  |  |  |  |  |
| AUC_0-24h_ (ng*h/ml) | 318 (50) | 126 (40) | 112 (57) | 45 (37) | 137 (17) | 136 (60) | 159 (36) | 290 (47) | 200 (46) | 122 (52) | 250 (53) |  |  |  |  |  |
|  |  |  |  |  |  |  |  |  |  |  |  |  |  |  |  |  |
|  | | | | | | |  |  |  |  |  |  |  |  |  |  |
| **Dacomitinib** | *Cycle 2 Day 1** | | | | | |  |  |  |  |  |  |  |  |  |  |
| *Mean (CV%)* | n = 6 | n = 3 | n = 5 | n = 2 | n = 3 | n = 5 | n = 1 | n = 2 | n=2 | n = 18 | n =11 |  |  |  |  |  |
| C_max_ (ng/ml) | 47.4 (71) | 26.3 (76) | 26.0 (44) | 25.7 (18) | 33.5 (47) | 37.3 (33) | 32.0 | 37.5 (44) | 55.9 (9) | 31.2 (49) | 51.7 (45) |  |  |  |  |  |
| t_max_ (h) | 7.6 | 11 | 9.6 | 2 | 5 | 4.4 | 8.0 | 4.5 | 4.5 | 7.2 | 4.8 |  |  |  |  |  |
| AUC_0-24h_ (ng*h/ml) | 888 (70) | 513 (75) | 549 (45) | 262 (11) | 654 (40) | 747 (43) | 647 | 658 (38) | 789 (44) | 616 (48) | 909 (50) |  |  |  |  |  |
|  | | | | | | |  |  |  |  |  |  |  |  |  |  |
| **PD-0325901** | *Cycle 1 Day 1* | | | | | |  |  |  | *All 6 mg*  *doses* |  |  |  |  |  |  |
| *Mean (CV%)* | n = 6 | n = 4 | n = 6 | n = 2 | n = 3 | n = 8 | n = 3 | n = 3 | n = 4 | n = 14 |  |  |  |  |  |  |
| C_max_ (ng/ml) | 68.7 (32) | 43.7 (59) | 85 (29) | 106.5 (5) | 179 (83) | 262 (30) | 192 (32) | 192 (32) | 134 (80) | 227 (43) |  |  |  |  |  |  |
| t_max_ (h) | 1.2 | 1.3 | 1.2 | 2 | 1.4 | 1.4 | 1.3 | 1.3 | 1.5 | 1.5 |  |  |  |  |  |  |
| AUC_0-12h_ (ng*h/ml) | 188 (20) | 160 (20) | 273 (15) | 452 (46) | 552 (55) | 822 (32) | 635 (17) | 635 (17) | 463 (63) | 706 (41) |  |  |  |  |  |  |
|  | | | | | | |  |  |  |  |  |  |  |  |  |  |
| **PD-0325901** | *Cycle 2 Day 1** | | | | | |  |  |  |  |  |  |  |  |  |  |
| *Mean (CV%)* | n = 6 | n = 3 | n = 5 | n = 2 | n = 4 | n = 5 | n = 1 | n = 2 | n = 2 | n = 6 |  |  |  |  |  |  |
| C_max_ (ng/ml) | 58.3 (28) | 47.7 (38) | 73.8 (51) | 125 (61) | 229 (68) | 242 (27) | 254 | 56 (17) | 155 (23) | 217 (25) |  |  |  |  |  |  |
| t_max_ (h) | 1.5 | 1.1 | 2.2 | 1 | 1 | 1.2 | 1 | 4.5 | 2 | 2.2 |  |  |  |  |  |  |
| AUC_0-12h_ (ng*h/ml) | 210 (10) | 145 (92) | 279 (21) | 376 (33) | 638 (65) | 855 (35) | 1341 | 406 (24) | 667 (33) | 954 (36) |  |  |  |  |  |  |

** Samples taken on cycle 2 day 1 or for intermittent dosing on the last day of concomitant use of both drugs*
