# Supplementary figures and images for: Phase 1 study of the pan-HER inhibitor dacomitinib plus the MEK1/2 inhibitor PD-0325901 in patients with KRAS-mutation-positive colorectal, non-small-cell lung and pancreatic cancer
Source: Br J Cancer. 2020 Mar 9;122(8):1166–74. doi: 10.1038/s41416-020-0776-z (PMC7156736; doi:10.1038/s41416-020-0776-z)

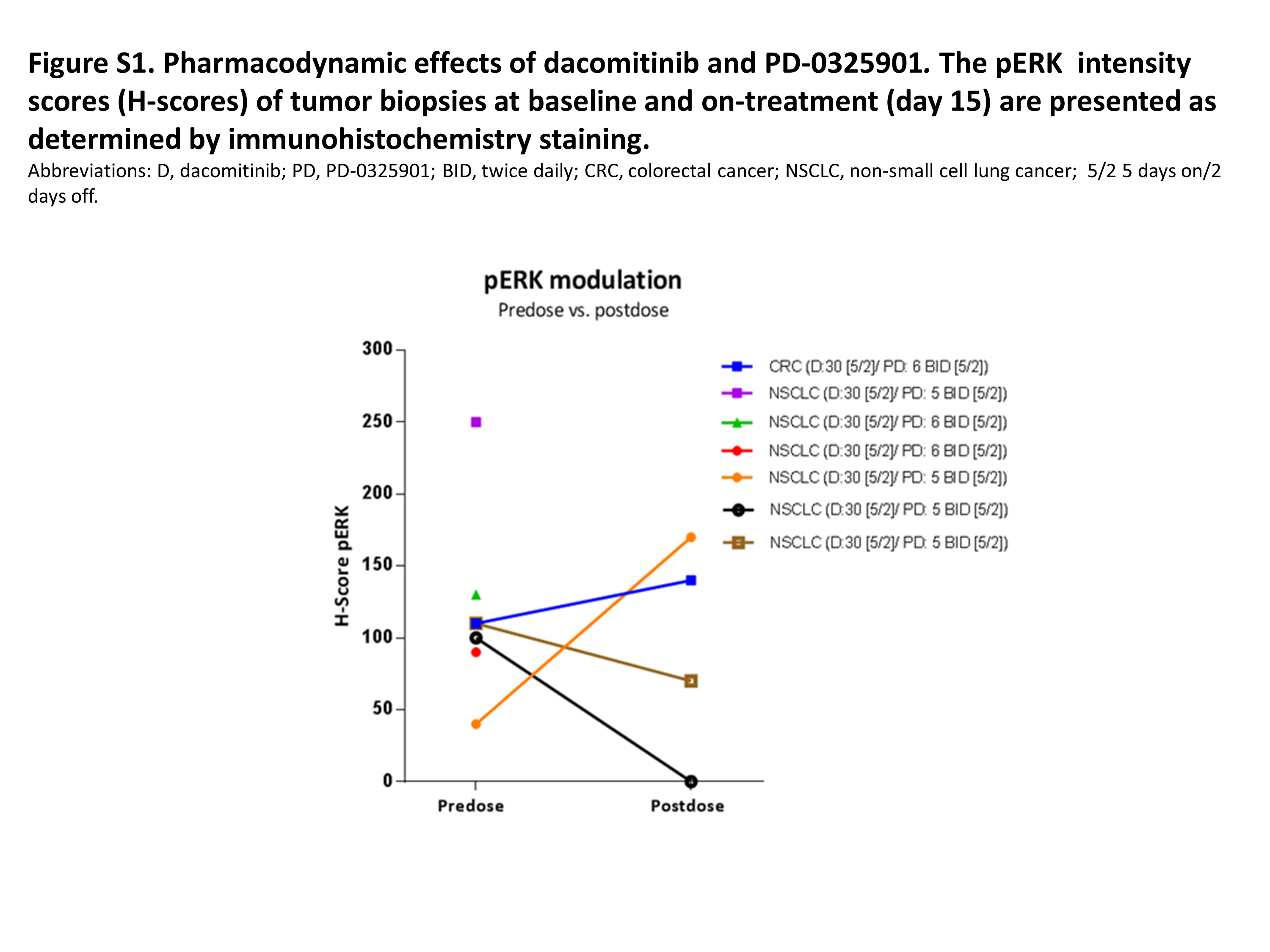

Supplement: Supplementary file 3 — Figure S1 [file 41416_2020_776_MOESM3_ESM.tif]
